# Comparative Analysis of Antimicrobial Properties and Physical Properties of Mineral Trioxide Aggregate Mixed With Distilled Water, Chlorhexidine, and Sodium Hypochlorite Solutions: An In Vitro Study

**DOI:** 10.7759/cureus.67563

**Published:** 2024-08-23

**Authors:** Balaji Suresh, Vignesh Ravindran

**Affiliations:** 1 Department of Pediatric and Preventive Dentistry, Saveetha Dental College and Hospitals, Saveetha Institute of Medical and Technical Sciences, Saveetha University, Chennai, IND

**Keywords:** mineral trioxide, compressive strength, antimicrobial property, sodium hypochlorite, chlorhexidine

## Abstract

Background: Endodontics widely uses mineral trioxide aggregate (MTA) because of its excellent sealing ability, biocompatibility, and capacity to promote healing. However, the effectiveness of MTA can vary depending on the blending solution used. Endodontics commonly employ chlorhexidine (CHX) and sodium hypochlorite (NaOCl), but their impact on MTA's properties necessitates further investigation.

Materials and methods: We blended MTA with the specified solutions and prepared it for testing according to the manufacturer's instructions. The study was divided into four groups: group 1 involved MTA blended with distilled water, group 2 consisted of MTA blended with 0.12% CHX solution (PerioGard, Colgate-Palmolive, Osasco, Brazil), group 3 included MTA blended with 0.2% CHX solution (Corsodyl, GlaxoSmithKline Consumer Healthcare, England, UK), and group 4 comprised MTA blended with 5% NaOCl (Azure Research Lab Pvt. Ltd., New Delhi, India). The antimicrobial activity of each group was assessed using the agar diffusion method against *Enterococcus faecalis*, *Candida albicans*, and *Streptococcus mutans*. We measured the compressive strength at 1, 3, 7, and 21 days using an Instron universal testing machine (Hounsfield Test Equipment, Redhill, UK). Statistical significance was evaluated through one-way ANOVA and Kruskal-Wallis tests, with p values <0.05 considered significant.

Results: Group 3 (MTA blended with 0.2% CHX) exhibited the highest antimicrobial efficacy, with significantly larger inhibition zones against *Enterococcus faecalis* (25.25 ± 0.21 mm vs. 13.33 ± 0.12 mm, p = 0.011), *Candida albicans* (29.58 ± 0.24 mm vs. 16.97 ± 0.16 mm, p = 0.004), and *S. mutans* (26.37 ± 0.15 mm vs. 14.55 ± 0.25 mm, p = 0.027). Group 4 (MTA blended with 5% NaOCl) showed the highest compressive strength at one and three days (p = 0.032 and p = 0.021, respectively), but by 21 days, group 2 demonstrated the greatest compressive strength (p = 0.044).

Conclusion: MTA mixed with 0.2% CHX provides superior antimicrobial properties, making it suitable for enhanced microbial control in endodontic treatments. Conversely, MTA mixed with 0.12% CHX offers optimal long-term compressive strength. These findings guide selecting MTA formulations to maximize performance based on clinical needs.

## Introduction

Mineral trioxide aggregate (MTA) cement has revolutionized modern dentistry with its superior sealing ability, biocompatibility, and potential to promote healing [1]. Initially, MTA was derived from building-grade Portland cement mixed with bismuth oxide and was first introduced commercially as ProRoot MTA in 1997 [2]. The original gray MTA, despite its effectiveness, caused tooth discoloration due to the presence of bismuth oxide and calcium silicate, leading to the development of white MTA. White MTA, with its lower iron content and less dark calcium aluminoferrite, aims to reduce discoloration while preserving its beneficial properties [3,4].

In pediatric endodontics, it is crucial to preserve primary teeth until the permanent teeth erupt and maintain immature permanent teeth in a healthy state. MTA has proven beneficial for these purposes, showing superior results in primary teeth pulpotomy compared to formocresol [5,6]. Its excellent sealing properties, resistance to dissolution, biocompatibility, and ability to form a dentin bridge make it an ideal material [7]. MTA is more effective than calcium hydroxide for pulp capping because it limits internal resorption in deciduous teeth. It is also preferred for direct pulp capping in developing permanent teeth [8,9].

Initially, people used chlorhexidine (CHX) as a broad-spectrum disinfectant [10]. Later, researchers discovered its effectiveness in inhibiting dental caries and reducing dental plaque. In the United States, CHX has been available by prescription since 1986 as a 0.12% CHX gluconate solution, marketed under names such as Peridex (3M ESPE, St. Paul, MN) and Periogard (Colgate-Palmolive, Osasco, Brazil) [11]. More recently, the PerioChip (Dexcel Pharma Technologies Ltd., Or Akiva, Israel), a biodegradable chip impregnated with CHX, has been utilized to treat periodontal defects, enhancing the outcomes of scaling and root planing [12]. Studies performed in the lab have shown that CHX can kill several microorganisms that are common in infected root canals. These microorganisms include *Staphylococcus aureus*, *Enterococcus faecalis*, *Streptococcus salivarius*, *Streptococcus pyogenes*, *Escherichia coli*, *Candida albicans,* and others [13].

Despite its widespread use and numerous benefits, MTA has limited effectiveness against *E. faecalis*, a persistent pathogen commonly associated with endodontic infections [14]. This study investigates the effects of incorporating various CHX and sodium hypochlorite (NaOCl) concentrations into MTA to address this limitation. We specifically focused on antimicrobial agents commonly available in standard dental practice settings. By evaluating these modifications, the aim is to enhance the antimicrobial efficacy of MTA, thereby improving its effectiveness in clinical endodontic treatments.

## Materials and methods

Study setting and ethical approval

We conducted this in vitro study at the scientific materials research facility of Saveetha Dental College and Hospitals, Saveetha Institute of Medical and Technical Sciences, Chennai, India. The Institutional Scientific Review Board Committee approved the experimental procedures, design, and materials used (approval number: SRB/SDC/PhD/Pedo/2023/12/062).

Preparation of experimental materials

Per the manufacturer's instructions, we prepared the MTA (white MTA, Angelus, Londrina, Brazil), maintaining a 3:1 powder-to-liquid ratio. The liquid component varied between the study groups: group 1: MTA blended with distilled water; group 2: MTA blended with 0.12% CHX solution (PerioGard); group 3: MTA blended with 0.2% CHX solution (Corsodyl, GlaxoSmithKline Consumer Healthcare, England, UK); and group 4: MTA blended with 5% NaOCl (Azure Research Lab Pvt. Ltd., New Delhi, India). The MTA powder was mixed with the designated liquid for each group until a homogeneous, thick, putty-like consistency was achieved.

Antimicrobial property testing

Antimicrobial studies selected the agar diffusion method for its simplicity and ability to produce clear, measurable results. Despite its limitations, such as variations in diffusion rates, it remains a widely accepted method for initial screening, offering a straightforward way to compare the antimicrobial activity of different materials under controlled conditions. The samples were assessed for antimicrobial efficacy against three American-type culture collection (ATCC) strains: *E. faecalis* (ATCC 29212), *Candida albicans *(ATCC 10231), and *Streptococcus mutans (*ATCC 35668). The ATCC strains were acquired from HiMedia Laboratory in Mumbai, India. A total of 20 Mueller-Hinton agar plates were prepared, with five plates designated for each material group. Following inoculation with the specified microorganisms, three wells (4 mm in depth and 5 mm in diameter) were created in each agar plate. On a glass slab, the materials were mixed and immediately introduced into the wells. The plates were incubated at 37°C and examined after 24 hours. Inhibition zones were measured using a digital caliper, and the data were then organized and analyzed statistically.

Compressive strength testing

To assess compressive strength, we followed the American National Standards Institute/American Dental Association No. 96 standard using stainless steel molds with a diameter of 4 mm and a height of 6 mm. MTA samples were prepared and mixed according to the previously outlined method [14]. The mixtures were then compacted into the molds using a mixing spatula and dental pluggers to ensure uniform density and reduce porosity. Glass slides were placed at both ends of the molds until the materials had been fully set. Following the setting, the samples were extracted from the molds and incubated at 37°C for 24 hours. 

**Figure 1:**
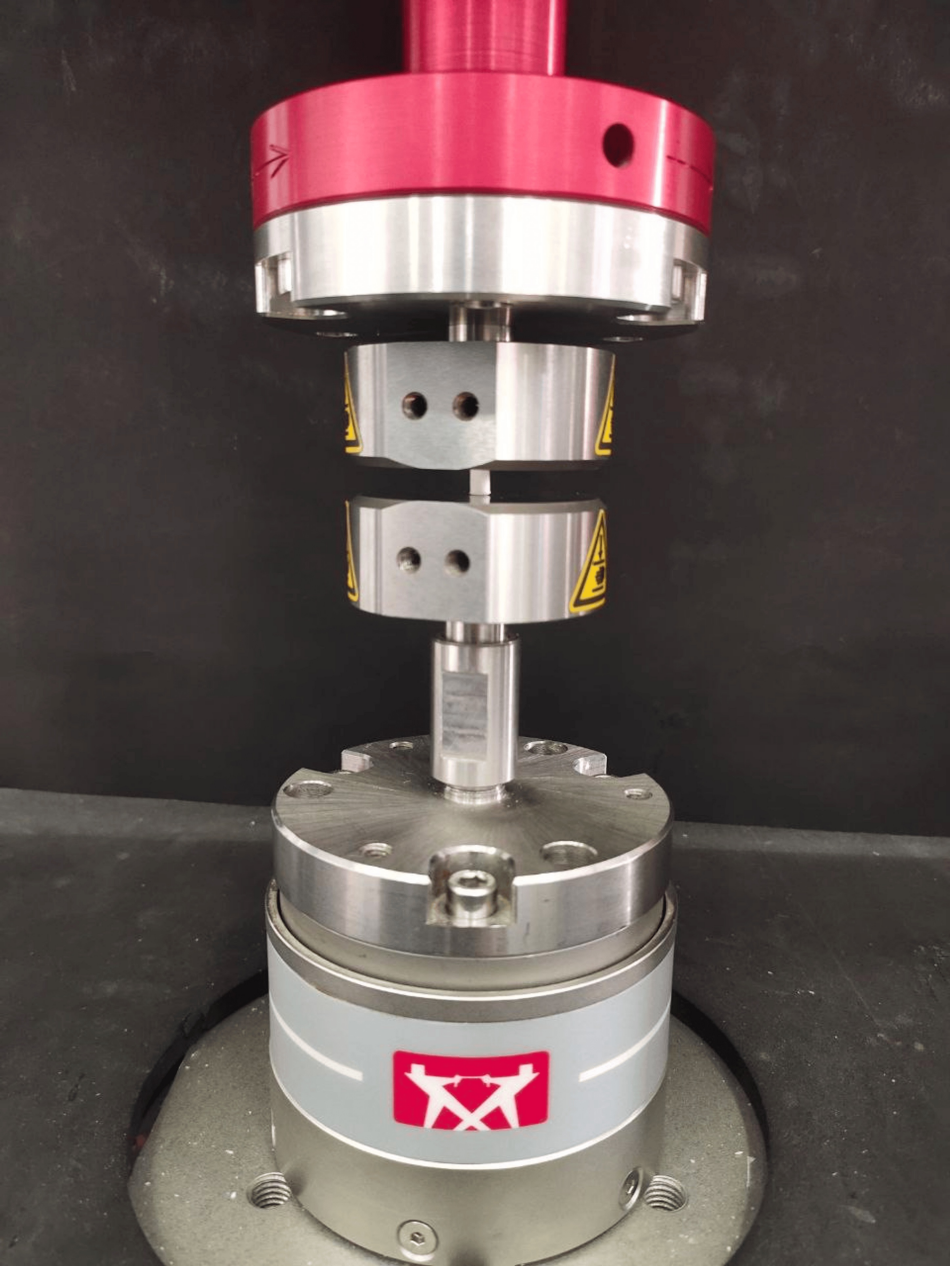
Material samples underwent a compressive strength test using the Instron universal testing machine

Forty specimens were prepared, with each group consisting of 10 samples. Compressive strength was measured using an Instron universal testing machine (Hounsfield Test Equipment, Redhill, UK) (Figure 1).

The specimens were subjected to a force applied parallel to the longitudinal axis of the molds at a crosshead speed of 1 mm/minute until failure. The maximum force at the fracture point was recorded in megapascals (MPa). The specimens were tested at the following time intervals: three hours, one day, three days, seven days, and 21 days after preparation. The collected data were then analyzed accordingly.

Data analysis

Data analysis was conducted using the IBM SPSS Statistics software, version 22 (IBM Corp., Armonk, NY). The Shapiro-Wilk test was employed to evaluate the normality of the data distribution. The Kruskal-Wallis test was applied to the inhibition zones to assess differences in antimicrobial efficacy. One-way ANOVA was utilized to compare compressive strength. Post hoc pairwise comparisons were carried out using Tukey's test. Statistical significance was determined with a p value threshold of <0.05.

## Results

Antimicrobial property

The antimicrobial activity of MTA mixed with different solutions was evaluated against three microorganisms: *E. faecalis*, *C. albicans*, and *S. mutans*. For *E. faecalis*, group 3 showed the greatest inhibition zone, measuring 25.25 ± 0.21 mm. This was followed by group 2 at 23.54 ± 0.16 mm, group 4 at 19.64 ± 0.22 mm, and group 1 at 13.33 ± 0.12 mm. For *C. albicans*, group 3 again demonstrated the highest antimicrobial activity with an inhibition zone of 29.58 ± 0.24 mm, followed by group 2 with 26.26 ± 0.19 mm, group 4 at 18.11 ± 0.21 mm, and group 1 at 16.97 ± 0.16 mm. In the case of *S. mutans*, the largest inhibition zone was observed in group 3 at 26.37 ± 0.15 mm, followed by group 2 at 24.76 ± 0.16 mm, group 4 at 20.26 ± 0.19 mm, and group 1 at 14.55 ± 0.25 mm.

Group 3 exhibited the highest antimicrobial efficacy against all tested microorganisms, suggesting its potential for enhanced microbial control in endodontic treatments. Conversely, group 1 showed the least antimicrobial activity across all tested groups. Table 1 shows a comparison of the zones of inhibition of all three tested materials against the three microorganisms.

**Table 1:** Comparison of the zones of inhibition of all three tested materials against the three microorganisms ^*^p <0.05 is statistically significant SD: standard deviation

Microorganisms	Material group	Mean ± SD (mm)	p value
E. faecalis	Group 1	13.33 ± 0.12	0.011^*^
	Group 2	23.54 ± 0.16	
	Group 3	25.25 ± 0.21	
	Group 4	19.64 ± 0.22	
C. albicans	Group 1	16.97 ± 0.16	0.004^*^
	Group 2	26.26 ± 0.19	
	Group 3	29.58 ± 0.24	
	Group 4	18.11 ± 0.21	
*S. mutans*	Group 1	14.55 ± 0.25	0.027^*^
	Group 2	24.76 ± 0.16	
	Group 3	26.37 ± 0.15	
	Group 4	20.26 ± 0.19	

The pairwise comparisons revealed that group 3 demonstrated the most significant antimicrobial activity against all tested microorganisms. Significant differences were observed between group 3 and other groups for *E. faecalis*, *C.* *albicans*, and *S. mutans*, except for some comparisons between groups 2 and 3 and groups 2 and 4, where differences were insignificant. Group 1 consistently showed the lowest inhibition zones across all microorganisms, indicating its relatively poor antimicrobial efficacy compared to the other groups. Table 2 shows pairwise comparisons of all three tested materials against the three microorganisms.

**Table 2:** Pairwise comparisons of the different test materials against different organisms ^*^p < 0.05 is statistically significant

Material (1-4)	Significance		
	*E. faecalis*	C. albicans	*S. mutans*
Group 1-2	0.01^*^	0.01^*^	0.01^*^
Group 1-3	0.01^*^	0.01^*^	0.01^*^
Group 1-4	0.01^*^	0.01^*^	0.01^*^
Group 2-3	0.12	0.26	0.18
Group 2-4	0.01^*^	0.01^*^	0.01^*^
Group 3-4	0.01^*^	0.01^*^	0.01^*^

**Figure 2:**
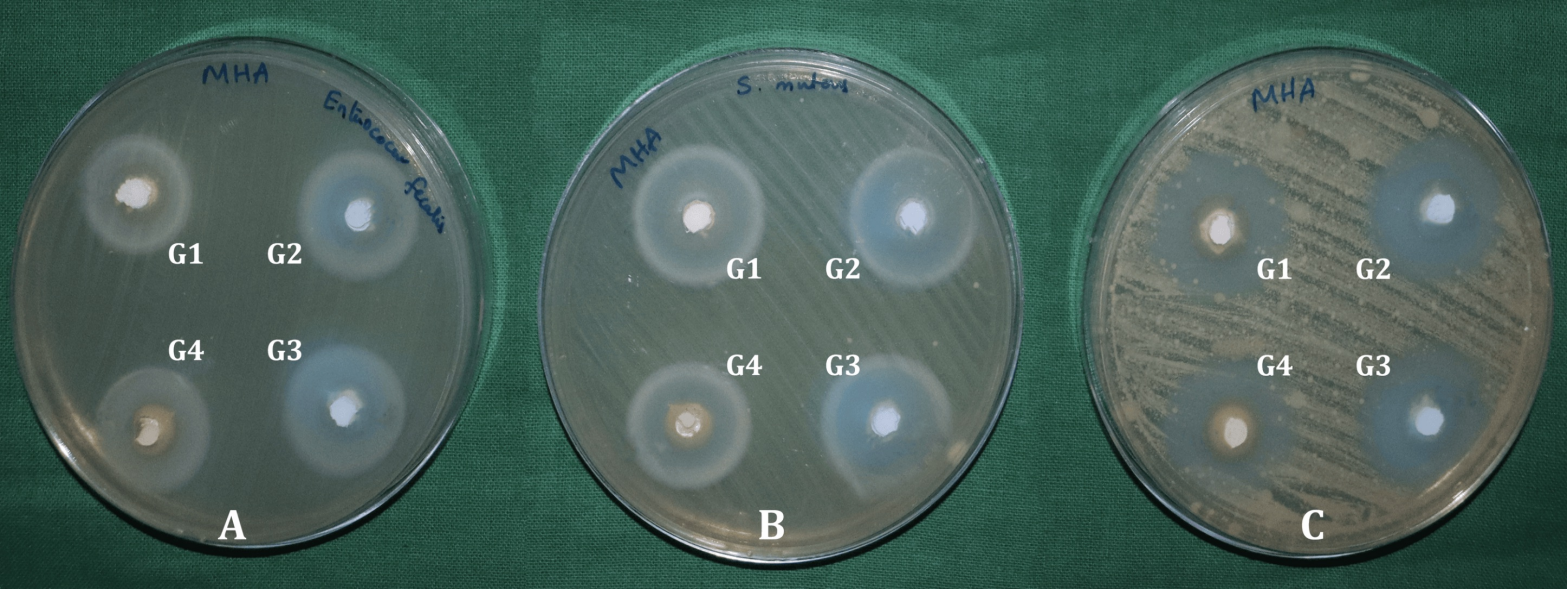
Agar well-diffusion method showing the zone of inhibition of the test materials against (A) E. faecalis, (B) S. mutans, and (C) C. albicans G1: MTA blended with distilled water; G2: MTA blended with 0.12% CHX solution; G3: MTA blended with 0.2% CHX solution; G4: MTA blended with 5% NaOCl MTA: mineral trioxide aggregate; CHX: chlorhexidine; NaOCl: sodium hypochlorite

Figure 2 shows the zone of inhibition of the tested materials.

Compressive strength

At three hours, none of the groups exhibited any compressive strength. By day 1, group 4 demonstrated the highest mean compressive strength at 90.93 ± 0.19 MPa, which was statistically significant compared to the other groups (p = 0.032). Groups 2 and 3 had the least compressive strength (87.39 ± 0.14 and 87.46 ± 0.23 MPa, respectively), while group 1 had a mean compressive strength of 89.24 ± 0.17 MPa. At three days, group 4 again showed the highest mean compressive strength at 102.14 ± 0.22 MPa, significantly higher than the other groups (p = 0.021). Group 1 recorded the least mean compressive strength of 95.85 ± 0.26 MPa. At seven days, group 2 displayed the highest mean compressive strength at 106.15 ± 0.29 MPa, followed by group 3 at 105.54 ± 0.14 MPa, group 4 at 104.31 ± 0.25 MPa, and group 1 at 103.48 ± 0.15 MPa. However, the differences among the groups were not statistically significant (p = 0.123). By 21 days, group 2 had the highest mean compressive strength at 160.25 ± 0.15 MPa, followed closely by group 3 at 159.37 ± 0.11 MPa. Group 1 recorded 152.93 ± 0.08 MPa, while group 4 showed the lowest mean compressive strength at 149.15 ± 0.14 MPa. The differences observed at this time point were statistically significant (p = 0.044).

Table 3 shows the comparison of the mean compressive strength of the different test materials at different periods of three hours, one day, three days, seven days, and 21 days.

**Table 3:** Comparison of the mean compressive strength of the different test materials at different periods of three hours, one day, three days, seven days, and 21 days ^*^p < 0.05 is statistically significant SD: standard deviation; MPa: megapascals

Period	Mean compressive strength ± SD (MPa)				p value
	Group 1	Group 2	Group 3	Group 4	
3 hours	0	0	0	0	-
1 day	89.24 ± 0.17	87.39 ± 0.14	87.46 ± 0.23	90.93 ± 0.19	0.032^*^
3 days	95.85 ± 0.26	98.73 ± 0.16	99.25 ± 0.17	102.14 ± 0.22	0.021^*^
7 days	103.48 ± 0.15	106.15 ± 0.29	105.54 ± 0.14	104.31 ± 0.25	0.123
21 days	152.93 ± 0.08	160.25 ± 0.15	159.37 ± 0.11	149.15 ± 0.14	0.044^*^

## Discussion

This study evaluated MTA's antimicrobial properties and compressive strength when blended with various solutions, including distilled water, 0.12% CHX, 0.2% CHX, and 5% NaOCl. The findings indicate that MTA mixed with 0.2% CHX exhibited the highest antimicrobial efficacy against all tested microorganisms. This result is consistent with the study by Ramezani et al. [15], which demonstrated that 0.12% CHX improved the sealing ability of apical plugs against salivary microbiota. MTA blended with 0.2% CHX produced significantly larger inhibition zones against *E. faecalis*, *C. albicans*, and *S. mutans* compared to MTA blended with distilled water [15]. In contrast, Bidar et al. reported no significant differences in antimicrobial properties among MTA blended with various concentrations of CHX (0.12%, 0.2%, and 2%) [16]. Additionally, Stowe et al. found that MTA blended with 0.12% CHX resulted in larger inhibition zones relative to MTA blended with distilled water, while Holt et al. observed enhanced antimicrobial effects against *E. faecalis* with 2% CHX. However, this was associated with reduced compressive strength [17,18].

In the study by Ravindran and Jeevananda [14], conventional MTA demonstrated significant antimicrobial activity against *E.* *faecalis*, *S. mutans*, and *C. albicans*. However, MTA modified with calcium fluoride exhibited notably superior antibacterial effects against these pathogens compared to its conventional counterpart. These findings align with our study, where MTA mixed with distilled water showed reduced antimicrobial efficacy compared to MTA combined with 0.2% CHX. This highlights the critical role of selecting an appropriate blending solution to optimize the antimicrobial performance of MTA. The superior antimicrobial activity observed in MTA blended with 0.2% CHX in our study reinforces the idea that incorporating specific antimicrobial agents can substantially enhance the effectiveness of MTA in endodontic treatments.

The variance in antimicrobial activity among MTA formulations mixed with different solutions can be attributed to several factors. Primarily, the concentration of the antimicrobial agent is crucial; MTA mixed with 0.2% CHX demonstrated enhanced antimicrobial efficacy compared to 0.12% CHX due to the increased concentration of the active compound. NaOCl, while a potent antimicrobial agent, may interact with MTA components, potentially altering its setting properties or pH, thus impacting its antimicrobial performance. CHX, with its higher stability and efficacy at elevated concentrations, provides sustained antimicrobial activity, whereas lower concentrations might not achieve sufficient effectiveness. Additionally, differences in microbial susceptibility to various antimicrobial agents contribute to the observed discrepancies in antimicrobial activity. Collectively, these factors elucidate why MTA mixed with 0.2% CHX exhibited superior antimicrobial properties compared to other formulations.

Regarding compressive strength, MTA blended with 5% NaOCl exhibited superior strength at one and three days. However, after 21 days, MTA blended with 0.12% CHX displayed the greatest compressive strength. This result contrasts with the findings of Bidar et al., who found that MTA blended with CHX had higher compressive strength than MTA mixed with distilled water. However, this difference did not reach statistical significance (p > 0.05) [19]. Our study corroborates the observation that while MTA mixed with 5% NaOCl achieves greater initial strength, MTA mixed with 0.12% CHX offers superior compressive strength over a longer period.

MTA blended with 0.2% CHX provides the best antimicrobial performance, though it does not always differ significantly from 0.12% CHX and NaOCl in some comparisons. Therefore, MTA formulations with 0.2% CHX are optimal for enhanced microbial control, whereas those with 0.12% CHX are preferable for maintaining structural integrity over time.

This study elucidates the antimicrobial efficacy and compressive strength of MTA mixed with different solutions, yet translating these findings into clinical practice necessitates further investigation into the biocompatibility of these formulations. Additional research should focus on conducting comprehensive biocompatibility and toxicity assessments to ensure that these MTA mixtures are safe for human use. Specifically, in vitro cytotoxicity assays and in vivo biocompatibility studies are essential to evaluate the potential adverse effects of these mixtures on human tissues. These studies will provide critical data on the safety profile of the MTA formulations, thereby informing their clinical applicability. A rigorous biocompatibility evaluation must confirm that the observed antimicrobial and mechanical benefits do not compromise patient safety or tissue health.

## Conclusions

This investigation evaluated the antimicrobial efficacy and compressive strength of MTA when blended with different solutions: distilled water, 0.12% CHX, 0.2% CHX, and 5% NaOCl. The results demonstrated that MTA blended with 0.2% CHX exhibited the highest antimicrobial efficacy against *E. faecalis*, *C. albicans*, and *S. mutans*. This superior antimicrobial performance makes MTA blended with 0.2% CHX the preferred choice for enhanced microbial control in endodontic treatments. Regarding compressive strength, MTA blended with 5% NaOCl showed the highest strength at initial time points, while MTA blended with 0.12% CHX displayed the greatest compressive strength at 21 days. Consequently, MTA blended with 0.2% CHX is recommended for its antimicrobial advantages, whereas MTA blended with 0.12% CHX is suggested for its optimal long-term compressive strength. These findings offer valuable guidance for selecting MTA formulations to meet specific clinical requirements.
